# Wake-Up Stroke and Stroke of Unknown Onset: A Critical Review

**DOI:** 10.3389/fneur.2014.00153

**Published:** 2014-08-12

**Authors:** Anke Wouters, Robin Lemmens, Patrick Dupont, Vincent Thijs

**Affiliations:** ^1^KU Leuven Department of Neurosciences and Experimental Neurology, KU Leuven, Leuven, Belgium; ^2^Department of Neurology, University Hospital Leuven, Leuven, Belgium; ^3^Medical Imaging Research Center, UZ Leuven, Leuven, Belgium; ^4^Leuven Research Institute for Neuroscience and Disease (LIND), KU Leuven, Leuven, Belgium; ^5^Laboratory of Neurobiology, Vesalius Research Center, Leuven, Belgium; ^6^Laboratory for Epilepsy Research, KU Leuven, Leuven, Belgium; ^7^Laboratory for Cognitive Neurology, KU Leuven, Leuven, Belgium

**Keywords:** wake-up-stroke, unknown-onset stroke, thrombolysis, thrombectomy, circadian rhythm

## Abstract

Patients, who wake up with an ischemic stroke, account for a large number of the total stroke population, due to circadian morning predominance of stroke. Currently, this subset of patients is excluded from revascularization-therapy since no exact time of onset is known. A large group of these patients might be eligible for therapy. In this review, we assessed the current literature about the hypothesis that wake-up-strokes occur just prior on awakening and if this subgroup differs in characteristics compared to the overall stroke population. We looked at the safety and efficacy of thrombolysis and interventional techniques in the group of patients with unknown stroke-onset. We performed a meta-analysis of the diagnostic accuracy of the diffusion-FLAIR mismatch in identifying stroke within 3 and 4.5 h. The different imaging-selection criteria that can be used to treat these patients are discussed. Additional research on imaging findings associated with recent stroke and penumbral imaging will eventually lead to a shift from a rigid time-frame based therapy to a tissue-based individualized treatment approach.

## Introduction

About one in six persons older than 45 will suffer from stroke in their remaining lifetime (1). The only Food and Drug Administration approved medical therapy for stroke is intravenous tissue plasminogen activator (alteplase or IV tPA), which should be administered preferably as quickly as possible within 4 h 30 min (2). Despite the efficacy of IV tPA, the narrow therapeutic window precludes wide scale use of the therapy mainly because a large majority of patients arrive too late in the hospital. Additionally, observational studies indicate that between 8 and 25% of patients come to the emergency room with an unknown time of symptom onset (3–7). This includes patients who were sleeping and woke up with stroke-symptoms or patients who are unable to state the time of symptom onset and in whom no witness is available. Theoretically, if patterns on cerebral imaging could serve as a substitute for the time since the stroke had occurred, then this subset of stroke patients would not be excluded from thrombolytic therapy. Another approach could be to rely on the presence of imaging characteristics indicative of large areas of viable tissue, regardless of the individual’s time since symptom onset, and use this information to use thrombolysis or other treatment strategies.

A large randomized controlled trial is currently being conducted in Europe to test MRI-based thrombolysis in strokes of unknown onset (8). The approach is to select patients based on an imaging pattern that appears to substitute reasonably well for time since stroke onset, the so-called diffusion/FLAIR (DWI/FLAIR) mismatch.

Here, we review the literature on “strokes of unknown onset” or that occur on awakening. We moreover provide a detailed overview of treatment studies that have already been completed in wake-up-strokes.

## Materials and Methods

In this paper, we review the evidence supporting the hypothesis that strokes discovered on awakening are recent. We assess the current literature on the efficacy and safety of thrombolysis in wake-up-stroke patients and patients with unknown onset of stroke. Finally, we review the imaging techniques that are proposed to determine if a patient with unknown onset of stroke will benefit from thrombolytic therapy.

A single author searched for articles in the Pubmed and Embase bibliographic databases using the following search terms: “wake-up-stroke,” “unknown-onset stroke,” and both these terms with the additional term ‘treatment,’ “circadian variation and stroke,” and “diffusion-flair mismatch.” We restricted our search on articles between 1990 and May 2014. We reviewed articles in the reference lists of included articles. We restricted our search to articles published in English. The final reference list was generated on the basis of relevance to the topics covered in this review. We performed a meta-analysis of diagnostic studies that assessed the DWI/FLAIR mismatch pattern in relationship to time since onset in unselected patients with precisely known symptom onset. We excluded articles that only focused on posterior circulation stroke or in whom sensitivity and specificity could not be determined from the provided information. Two of the authors (AW, VT) independently extracted the number of true positives, false positives, true negatives, and false negatives within the first 3 h and within the first 4.5 h after symptom onset of each of the included studies. Discrepancies between the two authors were resolved by consensus. Data analysis was conducted using the statistical program Stata (Version 12.0, StataCorp, College Station, TX, USA), and the user-written command-midas, a module for meta-analytical integration of diagnostic test accuracy studies (author Ben Dwamena, Division of Nuclear Medicine, Department of Radiology, University of Michigan Health System, Ann Arbor, USA).

## Results

### Circadian variation in stroke onset

Similar to acute myocardial infarction and sudden cardiac death, there is a diurnal variation in the onset of stroke, with a higher frequency of strokes occurring in the morning. The incidence of early-morning strokes rises with around 50% compared to the nightly incidence (9). This variation is seen regardless of the type of stroke (ischemic, hemorrhagic, and transient ischemic attacks) in some publications (10), but other studies suggest a tendency to a bimodal curve in hemorrhagic strokes, with a second peak in the afternoon (11).

The mechanisms underlying this diurnal variation in cerebrovascular events are not exactly known. Endogenous factors may play a role in this early-morning dominance in cardiovascular events. An increase in blood pressure, an increase in platelet aggregation, and a peak in prothrombotic factors are thought to be contributing factors (12–14). Blood pressure is typically lower during the night and increases upon awakening (12). This phenomenon is prone to individual variation with some people having an exaggerated response (15). This so-called “morning surge” in blood pressure, is an independent risk factor for stroke. It is speculated that the blood pressure leads to an increase in the likelihood of the rupture of a fragile atherosclerotic plaque. Timing the administration of anti-hypertensive medication in the evening has been proposed as strategy to circumvent the early-morning rise (16). A morning increase in platelet aggregation is mainly seen on arising and standing, and is probably due to an increase in catecholamine levels, platelet count, and hemo-concentration in the morning (14). An increase in the platelet adhesiveness in morning hours has been reported instead of increased platelet counts but this may be caused by different measurement-techniques (17). Furthermore, Kozinski et al. (18) examined the diurnal effect of clopidogrel on the inhibition of platelet aggregation. They found less inhibition in the morning hours. A small study (*n* = 11) showed an increase in Lp(a) and fibrinogen, during the morning hours (19). It is not well understood how these molecules contribute to acute cardiovascular events, apart from their effect on chronic atherosclerosis. A matutinal endothelial dysfunction has also been reported. Using high-resolution ultrasound of brachial artery flow-mediated dilatation, a blunting of endothelial function in the morning was found (20). Integrity of endothelial function is important for several homeostatic mechanisms that influence cardiovascular risk and in this way might contribute to acute cerebrovascular events. But again, no consistent results were found (21, 22).

**Figure 1:**
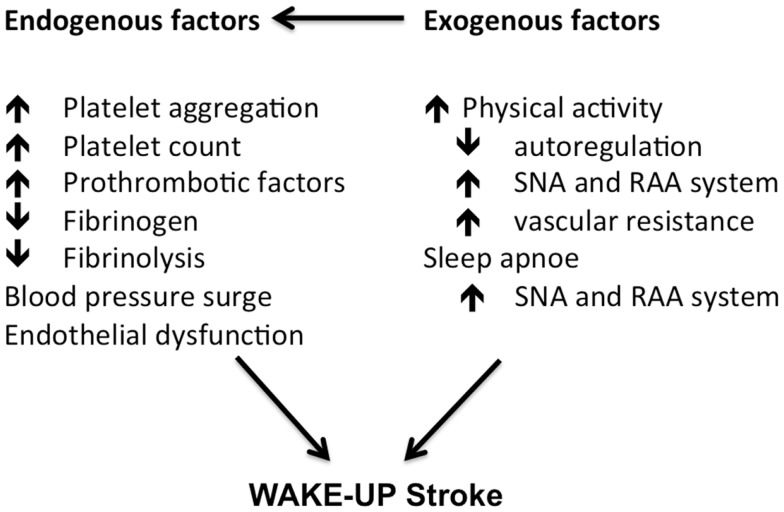
**Morning changes in cardiovascular factors contributing to a higher risk of stroke in the morning hours**. Adapted from Ref. (23).

Exogenous factors can play an additional role. The variation in circulatory factors can be a consequence of an early-morning response to arousal and physical activity in the awakening state (23). Data suggest a different response to exercise in the morning, with a blunting of the normal blood pressure lowering effects of exercise. Additionally, an association was found between the very common obstructive sleep apnoe syndrome (OSA) and the occurrence of wake-up-strokes (24). OSA is associated with intermittent hypoxemia and sympathetic overactivity, which increases the cardiovascular risk profile and possibly the prevalence of wake-up-strokes as well (Figure 1).

### Wake-up-stroke characteristics

The increase in stroke-occurrence in the morning has implications for possible treatment of patients with wake-up-stroke. If most of the strokes detected on awakening indeed occur in the early-morning hours just prior to awakening, the patients might be eligible for treatment. A recent epidemiologic study in the United States (7) found that 35.9% of wake-up-stroke patients would have been eligible for thrombolysis if arrival time were not a factor. To confirm or reject the hypothesis that wake-up-strokes occur early in the morning and have similar characteristics as strokes while awake, studies have been conducted to explore the possible differences between strokes detected on awakening and strokes while awake. Several studies report that the clinical and imaging characteristics do not differ between these groups (Table 1). One study (25) looked at the characteristics in terms of clinical impairment, NIHSS, age, gender, stroke subtype of patients with wake-up-stroke, and found no difference to a control-group with defined onset time. Functional outcome also was similar. Another study compared the clinical and imaging data of patients with known time of stroke-onset and those with wake-up-strokes, evaluated within 24 h after the moment, they were last seen normal (3). Both groups had similar perfusion (PWI) and DWI-lesion volumes on magnetic resonance imaging and a similarly high percentage of PWI/DWI mismatch, even in the subgroups of people within 3 h after detection. A CT-based imaging study (6) found no difference in percentage of CT perfusion CBF/CBV mismatch in 170 of the evaluated patients. Interestingly, patients with unknown time of onset occurring outside of awakening were also included, but this group had more neurological impairment and worse prognosis at discharge. Another study (26) found similar results with no differences in CT-findings between the known onset group and the wake-up group, but more hypodensities, indicative of later onset, were found in the unknown-onset group.

**Table 1:** **Clinical characteristic of patients with unknown-onset stroke**.

Differences between patients with known and unknown stroke						
Authors	Total number of patients (*n*)	Study set up and limitations	Wake-up (%) + unknown onset (%)	Clinical characteristics	Outcome	Imaging characteristics
Koton et al. (25)	4408	Prospective, consecutive, hospital based	19	N.s.d. (age, gender, TOAST, NIHSS)	N.s.d.	Not specified
Fink et al. (3)	364	Prospective, consecutive, hospital based	27	N.s.d. (age, gender, TOAST, NIHSS)	Not specified	DWI/PWI mismatch (*p* = 0.4): n.s.d.
Silva et al. (6)	676	Prospective, hospital based (*n* = 2), consecutive. Not specified if reader was blinded, each center had own reader, time of detection was randomly chosen at 7.30 AM	20 + 18	N.s.d. (age, gender, TOAST, NIHSS); Unknown-onset patients were older (*p* = 0.08), more likely to be female (*p* = 0.04), and higher NIHSS (*p* < 0.01)	Not specified	CT perfusion CBF/CBV mismatch: n.s.d.
Serena et al. (27)	654	Retrospective, hospital based (*n* = 6), consecutive, three independent and blinded readers	24	Nsd. (age, gender, TOAST, NIHSS, aHT)	Not specified	Early signs of ischemia: n.s.d (*p* = 0.35)
Todo et al. (26)	81	Retrospective, consecutive, hospital based, only cardioemboligenic strokes, readers not specified	21 + 22	N.s.d (age, gender, TOAST, NIHSS)	Not specified	CT: more hypodens zones in unknown-onset group (*p* > 0.001)
Nadeau et al. (5)	2585	Prospective, consecutive, population based, informed consent, also inclusion of hemorrhagic strokes	13	More smokers (*p* = 0.0016), high blood pressure (*p* = 0.0144), and less tPa (2.1 vs. 13.5%)	Worse outcome: SIS seven points lower (*p* = 0.0012)	Not specified
Jimenez et al. (28)	813	Prospective, consecutive, hospital based	16	More obesity (*p* = 0.058), less tPa (0 vs. 3%)	Tendency to worse outcome (*p* = 0.038)	Not specified
Mackey et al. (7)	1854	Retrospective, hospital based (*n* = 7)	14.3	N.s.d. (age, gender, TOAST, NIHSS)	N.s.d.	Not specified

*N.s.d = no significant difference, TOAST = trial of org 10172 in acute stroke treatment, aHT = arterial hypertension, SIS = stroke impact scale*.

In contrast, some studies did report differences between wake-up strokes and strokes with defined onset. More severe neurological impairment and a non-significant trend toward worse functional outcome were observed in one study (28). Worse functional outcome, measured with the stroke impact scale (SIS-16) and a lower return to home after stroke was reported in a study that included hemorrhagic strokes. Worse outcomes were especially observed in subarachnoid hemorrhages occurring on awakening (5).

Although most studies report similar characteristics between strokes occurring at wake up and the strokes with a defined onset, these studies have limitations. Many studies were retrospective (7, 26, 27), single center (3, 25, 26, 28), and not population based (3, 6, 7, 25–28). Studies were heterogeneous in term of study population with some studies including hemorrhagic strokes (5). The inclusion and exclusion criteria varied across studies. Various imaging techniques were used and interpretation was not always blinded or performed by independent observers. Furthermore, publication bias cannot entirely be excluded, although this is unlikely since most of the studies are negative (i.e., showing no difference).

Further, prospective, population based, multicenter studies, using strictly defined inclusion criteria, employing standardized imaging with blind evaluation are therefore warranted.

### Medical and interventional therapy for patients with unknown time of stroke-onset: current evidence

We identified 12 studies in which wake-up-stroke-patients (WUS) were treated with intravenous tPA, either with or without intra-arterial therapy (Table 2). In 2006 (29) and 2008 (30) already, two case-reports were published about thrombolysis and mechanical reperfusion of patients with unknown stroke-onset. They suggested an imaging-based selection to define which patients would benefit from thrombolysis regardless of the time of stroke-onset.

**Table 2:** **Off-label treatment of patients with unknown-onset stroke**.

Authors	Design	Number of patients with wake up or unknown-onset stroke control-group	Mean NIHSS	Mean age (years)	Door to needle time (min)	Type of stroke + time from symptom recognition till treatment	Imaging criteria	Treatment	sICH (%)	mRs 0–1 (%)	mRs 0–2 (%)
Iosif et al. (30)	Case-report	2	–	–	–	Wake-up	DWI/PWI and DWI/FLAIR mismatch	Intra-arterial tPa, thrombectomy			
Cho et al. (31)	Retrospective, observational three centers	32 223 (known onset)	14.5	67.5	154	Wake-up/unknown onset + 3–6 h	DWI/PWI –and DWI/FLAIR mismatch + DWI <1/2 ACM	IV tPa, IV tPa, and intra-arterial urokinase, intra-arterial urokinase	6.3	37.5	50
Adams et al. (32) AbESTT	Randomized clinical trial, >20 centers	22 (treatment) 21 (placebo) 758 (known onset)	10	68.6	?	Wake-up + <3 h	NCCT, CT < 1/2 ACM	Abciximab	13.6	10	32
Barreto et al. (4)	Retrospective, observational, 1 center	46 (treatment) 34 (no treatment) 174 (known onset)	16 (treatment) 10 (no treatment)	62.0	144	Wake-up + no further time specification	NCCT, CT > 1/3 ACM	IV tPa, IV tPa, and intra-arterial urokinase, intra-arterial urokinase	4.3	14	28
Breuer et al. (33)	Prospective, 1 center	10 (treatment) 35 (no treatment)	10.5 (treatment) 6 (no treatment)	68	80	Wake-up + <6 h	MRI visual PWI/DWI mismatch, no FLAIR hyperintensity, no DWI > 1/3 MCA	IV tPa (0.9 mg/kg)	0	31	60
Kim et al. (34)	Retrospective, 1 center	29 (treatment) 49 (no treatment)	13	66.9	?	Wake-up/unknown onset, <3 h	NCCT, CT < 1/3 ACM + Consecutive PWI before intra-arterial tPa	IV tPa, IV tPa, and intra-arterial urokinase, intra-arterial urokinase	10.3	37.6	44.8
Aoki et al. (35)	Prospective, 1 center	10	14	84	?	Wake-up/unknown onset, <3 h	DWI/FLAIR mismatch	IV tPa (0.6 mg/kg)	0	30	40
Ebinger et al. (36)	Observational substudy, 1 center	17 131 (known onset)	13	81	86	Wake-up/unknown onset + <24 h	MRI, DWI < 1/3 ACM	IV tPa (0.9 mg/kg)	0	29.4	41.2
Kang et al. (37)	Prospective, 6 centers	83 (treatment) 156 (no treatment)	14	67.5	155	Wake-up/unknown onset + <6 h	DWI/PWI and DWI/FLAIR mismatch DWI < 1/3 ACM	IV tPa, IV tPa, and intra-arterial urokinase, intra-arterial urokinase	3.6	28.9	44.6
Michel et al. (38)	Single-center, prospective, randomized, double-blinded, placebo-controlled, phase II study	6 (treatment) 6 (no treatment)	16	59	122	Wake-up/unknown onset + <2 h	CT perfusion (MTT and CBV)	IV tPa (0.9 mg/kg)	0	?	4 (66.6)
Manawadu et al. (39)	Retrospective, case control, 1 center	68 326 (known onset)	12	74	73	Wake-up + >4.5 h, <12 h	NCCT, CT < 1/3 ACM	IV tPa (0.9 mg/kg)	2.9	16	37
Bai et al. (40)	Prispective, single center	48 138 (known onset)	11	61	?	Wake up + <12 h	MRI: DWI/FLAIR or T2 mismatch.	IV tPa	2	55	?
Natarajan et al. (41)	Retrospective, 1 center	30 (wake-up not specified)	13	72	210	Wake-up/known onset + 8–23 h	CT perfusion, >30% CBV CT <1/3 ACM	Intra-arterial thrombolysis, mechanical thrombectomy, balloon anioplasty, intra-arterial thrombolysis + mechanical thrombectomy, eptifibatide	33.3	?	20
Burkart et al. (42)	Retrospective, 1 center	40 (five unknown onset)	18	75.4	151	Wake-up/unknown onset/known onset + timing not further specified	Exclusion if MTT > 50% or NCCT > 1/3 ACM	Mechanical thrombectomy (sometimes with intra-arterial tPa or IV tPa)	10	?	50
Stampfl et al (43)	Retrospective, observational, 1 center	19	17	73.7	–	Wake-up + timing not further specified	1. DWI/PWI > 50%, 2. CBV/TTP > 50%, + <1/3 MCA	Mechanical thrombectomy by stent-retriever-devices (sometimes with intra-arterial tPa pr IVtPa)	21.1	10	10
Jung et al. (44)	Prospective, 1 center	55 (wake-up) –, 22 (unknown onset) 782 (known onset)	15 (wake up) 18 (unknown onset)	61.9 (WUS), 63.5 (UOS)		Wake-up/unknown onset/known onset + <24 h	No stringent criteria, individual decision, PWI/DWI mismatch was assessed	Intra-arterial urokinase, mechanical thrombectomy, and intra-arterial urokinase, mechanical thrombectomy	3.7, 9.1	16.7, 23.8	37, 38.1

*sICH = symptomatic intracranial hemorrhage, WUS = wake-up-stroke, UOS = unknown onset, NCCT = Non-contrast enhanced CT, mRs = modified ranking score, sICH = symptomatic intracranial hemorrhage*.

*Barreto et al. (4): outcome-data were obtained at discharge, no long-term outcome available*.

### Thrombolysis

Subsequent studies compared outcome after treatment of known- and unknown-onset ischemic stroke. One of the first was a retrospective study among three centers, which included 32 patients with unknown-onset stroke and 223 controls (31). There was no difference in the outcomes of the two groups. The limitations of this retrospective study include the small number of patients and the imbalance in treatment methods among the two groups with more use of intra-arterial thrombolysis in the unknown-onset group. Images were not analyzed uniformly and like in most of the current studies, only one reader was used to interpret the images. In the AbESTT-trial (32) abciximab, a GPIIbIIIb receptor blocker was tested in a randomized controlled trial as a potential treatment of acute ischemic stroke. A subgroup of WUS patients who presented within 3 h after symptom recognition was also included. Unfortunately inclusion of this subgroup was stopped early, because of an unacceptably high rate of symptomatic intracranial hemorrhages (SICH). In the end, it was shown that abciximab did not improve outcome overall. WUS patients in this study exhibited significantly more signs of early ischemia on CT compared to the non-wake-up group, suggesting that these patients did not have very early stroke and had less chance to respond to reperfusion-therapies. Barreto et al. (4) retrospectively compared the outcomes of wake-up patients who were treated with intravenous tPA, intra-arterial urokinase or a combination of both, and those who did not. Non-contrast CT was used to exclude a hypodensity larger than 1/3 of the middle cerebral artery territory. Multimodal neuroimaging was not a part of their study-protocol. Despite a greater clinical impairment, they found a higher rate of good outcomes in the treated patients, however, at the expense of an increased mortality. Additionally, a comparison was made between wake-up-strokes and patients with known onset time who received thrombolysis within 3 h of symptom onset. Having adjusted for baseline differences in NIHSS, outcomes were similar. This retrospective study is limited by its lack of clinical or imaging selection criteria, the various treatment modalities, a possible selection bias and the relatively small sample size. In a similar study, thrombolysis in wake-up patients was significantly associated with a favorable outcome at 3 months (odds ratio = 6.842) (34). When a large diffusion-deficit on MRI was found, tPA-administration was stopped. This happened in 5 out of the 22 cases at various time points after tPA infusion started. This increased the heterogeneity in the study population and highlights the need for uniform pre-treatment imaging selection criteria. A small study (*n* = 10), without a control-group, showed a safe selection of patients based on DWI/FLAIR mismatch with no SICH occurring (35). However, the sample size was obviously small and a lower than currently accepted dose of tPA (0.6 mg/kg) was used. Also the time from last seen well till treatment was on average 5.6 h, which is lower than in most other studies. A similar observational study reported no SICH and no difference in outcome in 17 similarly selected patients (36). The study of Kang et al. (37) had multiple advantages over the previous ones. A large group of patients with unknown-onset time of stroke (*n* = 83) were included and they used well-defined clinical and imaging selection criteria. Only WUS-patients were treated who both had a DWI/PWI and a DWI/FLAIR mismatch. After adjusting for age, sex, and baseline NIHSS score, reperfusion therapy significantly increased the incidence of good clinical outcomes in unclear-onset stroke patients compared to a matched-cohort of untreated patients (odds ratio, 2.25). However, although the clinical inclusion criteria were similar, the control patients did not undergo the same stringent imaging selection criterion, which biases the findings of this study. Other limitations were the participation of two centers with no previous experience in MRI-based thrombolysis studies. Only 1 of the 10 patients treated in these centers, did have a good outcome. Although they used two MRI-based selection criteria, no pre-trial training was foreseen. Organization of a training course in advance could have increased the reproducibility of the image protocols.

### Mechanical thrombectomy

Four studies [(41–44); Table 2] examined the possible benefit of mechanical thrombectomy, all based on perfusion (CT or MRI) scans and clinical criteria to include patients. One study (43) included 19 patients with wake-up-strokes. They used stent-retriever-devices for mechanical thrombectomy. Compared to other studies with known onset stroke, a larger number of SICH were found and patients had less favorable outcome after 3 months. Another study (44) found no significant difference in outcome between known and unknown-onset stroke patients. However, selection of patients was based on individual decision making and various treatment techniques were used over the years.

### Summary

From these pilot-studies, we conclude that many patients with wake-up or unknown-onset stroke might be helped by revascularization-therapy with relative safety. There is more experience with intravenous treatment than with endovascular therapies. Limitations in using clinical databases are the possible bias in selection of patients, completeness of data, and retrospective determination of outcome. Moreover, publication bias cannot be excluded, as there are no small studies published, which show unfavorable results. Only two studies (32, 38) testing thrombolysis or thrombectomy were randomized. One was the AbESTT-trial (32), discussed previously and the other one was a small pilot-study with only 12 patients included (38). The imaging selection criteria used to select patients for treatment were not uniform, since most centers used individual decision making to treat this subset of patients. A large clinical randomized trial is therefore needed to confirm these preliminary results.

### Proposed imaging selection methods

**Figure 2:**
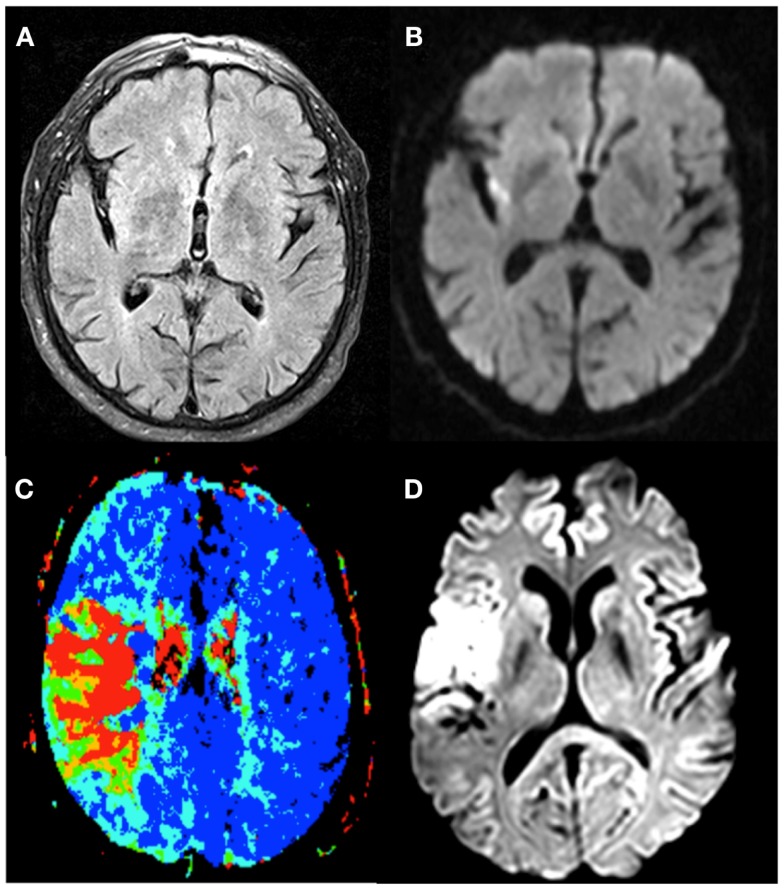
**The different imaging techniques used to select stroke-patients who would benefit from therapy**. Patient 1 exhibits no FLAIR-lesion **(A)** and a clear DWI-lesion **(B)**, the so-called DWI-FLAIR mismatch pattern. Patient 2 has a PWI/DWI mismatch on imaging, with **(C)** representing the lesion on Tmax, and **(D)** the corresponding diffusion lesion.

Unknown time of onset is clearly a major reason not to receive thrombolysis (45). In the studies with off-label treatment of unknown-onset stroke patients, different imaging selection criteria have been used [Table 2; (46)]. These include visual or semi-quantitative analysis of the FLAIR-DWI mismatch, PWI-DWI mismatch, or CT perfusion based approaches (Table 3; Figure 2).

**Table 3:** **Characteristics of the proposed imaging-modalities**.

	Advantages	Disadvantages
CT perfusion	• Widely available at ER • Fast • Low cost • Easy patient monitoring • Helps to identify patients who would benefit from therapy and those with high hemorrhagic risk	• Additional radiation dose • IV contrast • Difficult to detect small infarcts • Protocols and guidelines for quantitative thresholds vary • Different post-processing programs • AIF (arterial input function) and VOF (venous input function) difficult to localize • False positive results: Decreased blood flow due to vascular stenosis, extensive white matter disease, seizure and vasospasm • False negative results: partial volume effect around blood vessels
MRI perfusion	• High sensitivity and high predictive value for ischemia • Increasing evidence that ADC can reliable predict ischemic core • PWI/DWI mismatch for selection of patients who would benefit from therapy and those with high hemorrhagic risk (malignant profile)	• Duration of scan • Limited availability • Use of a contrast agent • Limitations: pacemakers, claustrophobia • Monitoring of patients is more difficult • Protocols and guidelines for quantitative thresholds vary • Different post-processing programs
DWI/FLAIR mismatch	• Imaging marker for timing of stroke-onset based on pathophysiologic tissue changes in the evolution of acute stroke • Qualitative assessment → no need for long postprocessing • Validation in large PRE-FLAIR study	• Relative high interrater- and intrarater-variability • Sensitivity is quite low → stroke-patients within time-interval to benefit from tPA can be missed

### DWI-FLAIR mismatch

Several groups used the presence of a DWI/FLAIR mismatch to guide thrombolysis in patients with unknown-onset stroke (33, 35, 37, 40). The idea behind the FLAIR-DWI mismatch analysis principle is based on the pathophysiology of acute cerebral ischemia. Diffusion-weighted imaging is most sensitive to a restriction of the Brownian motion of extracellular water caused by cytotoxic edema. This phenomenon is already present within minutes after the event. FLAIR-images are sensitive for the detection of vasogenic edema, a phenomenon, which starts gradually in the hours after the initial event. This edema is thought to reflect loss of the integrity of the blood brain barrier.

**Figure 3:**
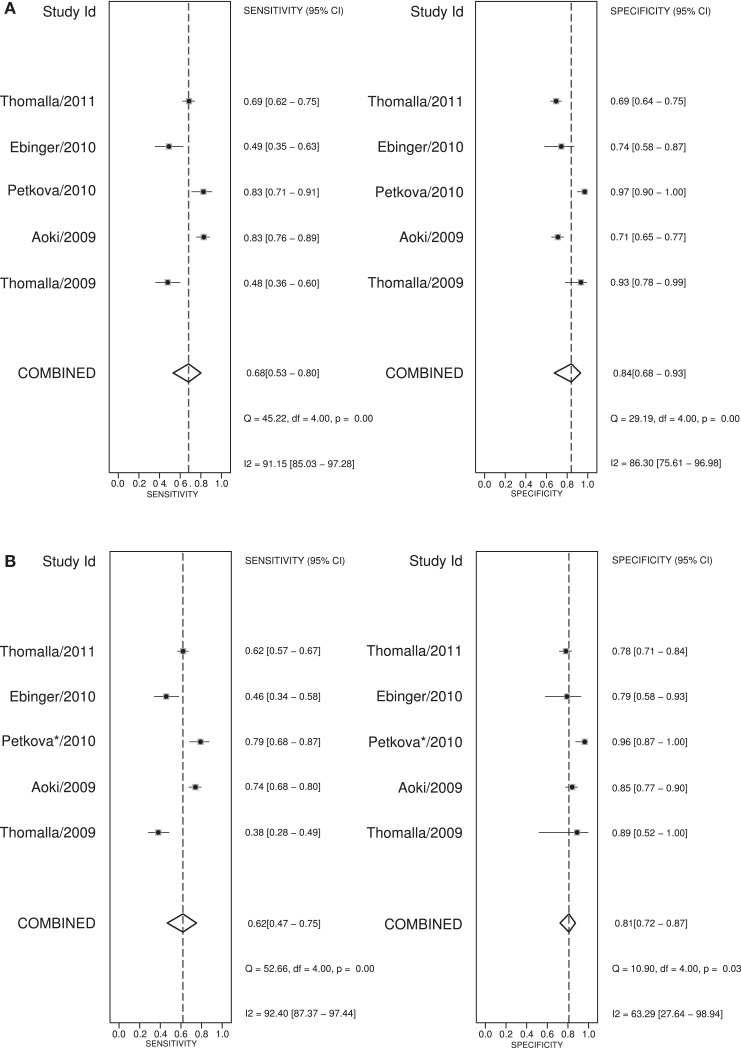
**Meta-analysis (47, 49–52) of the “strict” DWI/FLAIR mismatch pattern predicting stroke onset before 3 and 4 h**. **(A)** shows the sensitivity and specificity for predicting stroke-onset before 3 h and **(B)** shows these values for stroke-onset before 4.5 h. *To predict stroke onset before 4.5 h the “liberal” method of DWI/FLAIR mismatch was used in the study of Petkova et al. (49).

A large multicenter observational study showed that the pattern of an acute ischemic lesion seen with DWI but not with FLAIR-imaging, decreases with longer time between onset of symptoms and MRI scanning (47). DWI/FLAIR mismatch had a sensitivity of 62%, a specificity of 78%, and a positive likelihood ratio of 3.6, for the detection of stroke patients within 4.5 h of stroke onset. In that study, a visual analysis was used that required complete absence of even subtle FLAIR lesions. In a later substudy (48), a more liberal visual rating system was used, in which subtle FLAIR lesions were still considered as DWI/FLAIR mismatch. This improved the sensitivity (0.86), but decreased the specificity (0.48) of this pattern to detect lesions within 4.5 h. The positive likelihood ratio for the liberal rating system was 2.6. The disadvantage of the liberal rating system is an increase in the interrater-variability for “subtle” FLAIR lesions. Figure 3 shows a meta-analysis of reported diagnostic studies that assessed the DWI/FLAIR mismatch pattern in relationship to time since onset in patients with precisely known symptom onset. The overall sensitivity of the DWI/FLAIR mismatch for detecting stroke within 3 h is 74% and within 4.5 h is 62%, with a specificity of 82% at both time points. The diagnostic accuracy is quite heterogeneous and may reflect differences in inclusion criteria, definitions of FLAIR/DWI mismatch, acquisition techniques, proportions of small, and infratentorial lesions between the studies.

Semi-quantitative analysis of the FLAIR signal intensities has been proposed in order to avoid interpretation issues (53). However, this is controversial and in the PRE-FLAIR study an improvement over quantitative analysis of FLAIR signal was not better than visual analysis (48, 54). It has been shown that a significant amount of wake-up-stroke patients has such a DWI/FLAIR mismatch pattern (55). There are limitations though of the sole use of DWI/FLAIR mismatch to select patients for therapy. First there is no certainty about which technique (liberal or strict) has to be used to visually assess the presence of a DWI/FLAIR mismatch. Second, because of the low sensitivity, a large amount of possible eligible patients will be excluded from therapy. The interpretation of the mismatch pattern also depends on the type of stroke. With every 10 ml increase in diffusion volume, the odds to find a flair positive lesion increases with 7% (47). For patients with an infratentorial stroke, the DWI/FLAIR mismatch pattern seems to be less robust in identifying patients with early onset (56). The risk of bleeding in patients with a DWI/FLAIR mismatch is still uncertain. One recent study suggests a higher risk of bleeding with early FLAIR-hyperintensity (57), while another study found no association (58). Flair-positivity might be associated with a worse outcome after 3 months, but this finding still needs to be confirmed (59). Other imaging parameters have to be found to make a more optimal selection of patients who would benefit from therapy.

### CT perfusion

Another approach is to select patients based on the presence of imaging markers of tissue at risk or other imaging characteristics. CT combined with perfusion CT is a widely available technique that may help decide if patients with unknown-onset stroke, are eligible for off-label use of thrombolysis (29, 38, 41). Different hemodynamic parameters like cerebral blood volume (CBV), cerebral blood flow (CBF), delay time (Tmax), and mean transit time (MTT) have been proposed to identify areas of critical hypoperfusion. The ischemic core is variously defined as a region with markedly reduced CBV or CBF combined with prolonged MTT or Tmax. However, a real consensus on which parameter and threshold best represents critical hypoperfusion and core has not emerged yet. Different definitions with different thresholds have been proposed to define the tissue at risk. Wintermark et al. (60) did a ROC analysis and proposed an optimal threshold of 2 ml/100 g for CBV to define the ischemic core and 145% of MTT to define the tissue at risk of infarction. More recent evidence suggests that relative CBF might be better to define infarct core then CBV (61, 62). The more recent literature suggests a threshold of CT-Tmax of >6 s to define the tissue at risk (63). Despite that no real consensus exists about the thresholds that should be used, the speed and wide availability make perfusion CT an interesting alternative compared to other perfusion-modalities.

### PWI/DWI mismatch

Perfusion and diffusion based MR imaging techniques have been advocated as an imaging selection method in wake-up strokes (Figure 2). The disadvantage of the latter technique is the longer imaging time required and the limited availability of MRI compared to CT. Dynamic susceptibility contrast enhanced MRI is the most widely used technique. Arterial spin labeling is a newer method and has no need for contrast, but requires, in general, longer imaging times. MRI does have the advantage of a reliable prediction of the ischemic core with ADC-maps (64). However, the possible reversibility of the diffusion lesion, questions the paradigm that diffusion lesions represent the ischemic core. Analysis of the EPITHET-data showed that true DWI-lesion reversal is uncommon and if present would rarely alter treatment decision making (65). As with CT, difficulties arise in determining the optimal thresholds to differentiate ischemic core from salvageable brain tissue (66). Also the selection of the most optimal parameter or combination of parameters is still a matter of debate (67). An ADC-threshold of 600 × 10^−6^ mm^2^/s seems a fairly robust parameter in predicting ischemic core tissue (64). The mismatch between an area that has a Tmax > 6 s and is below this ADC-threshold is currently considered in several clinical trials as an operational definition of the tissue at risk. A substudy of DEFUSE 2 (68) supported this hypothesis by showing that in patients with a strong reperfusion, there is a high correlation between baseline DWI-volume and final infarct and in patients with minimal or no reperfusion, there is a high correlation between the baseline PWI-volume and final infarct. To determine the tissue at risk the PWI-DWI mismatch is useful, with 120% most commonly used to define a mismatch, although more stringent criteria have been advocated, with studies now advocating a perfusion-diffusion ratio that is larger than 180%, dependent on the parameter that is used to define the perfusion abnormality. MRI can also detect the so-called “ malignant” profile. The DEFUSE-data (69) showed that patients with a baseline DWI-lesion bigger than 100 ml and/or a PWI lesion of 100 ml or more with 8 s or longer of Tmax delay, suffered more intracranial hemorrhages after early reperfusion. The major drawback in the clinical use of MRI perfusion is that the extent of perfusion abnormalities varies among perfusion parameters, software packages, and various algorithms and that upon today no consensus is reached. Other techniques to define tissue at risk, like FDG-PET or SPECT, are less easily used in clinical practice. Two large trials to treat wake-up patients are currently ongoing, one based on DWI/FLAIR mismatch (WAKE-UP) (8) and one based on penumbral imaging (EXTEND) (70).

## Conclusion

Wake-up-stroke and stroke with unknown time of onset are frequent. These patients are at present excluded from thrombolytic therapy. Evidence suggests that these strokes occur closely on awakening and most observational studies did not find differences in terms of clinical features or outcome after therapy, suggesting that at least a subset of these patients could benefit of thrombolysis or endovascular treatment. Selection of eligible patients is preferably done using neuro-imaging, but the optimal imaging selection strategy for treating these patients has not yet been defined. The proposed selection modalities have not been properly evaluated in randomized trials, therefore, inclusion in these trials is primordial. In case randomization is not possible, advocating treatment on the basis of the presence of a DWI/FLAIR mismatch or PWI/DWI mismatch can generally not be recommended. In this scenario, treatment must remain an individualized decision. Ongoing randomized controlled trials testing these strategies are the WAKE-UP trial (8) and the EXTEND-trial (70). Identifying a safe and efficacious selection strategy will not only benefit patients with WUS, but also allow moving away from a rigid time window based approach for all patients.

## Conflict of Interest Statement

The authors declare that the research was conducted in the absence of any commercial or financial relationships that could be construed as a potential conflict of interest.
